# Hsa_circ_0001946 Inhibits Lung Cancer Progression and Mediates Cisplatin Sensitivity in Non-small Cell Lung Cancer via the Nucleotide Excision Repair Signaling Pathway

**DOI:** 10.3389/fonc.2019.00508

**Published:** 2019-06-12

**Authors:** Ma-Sha Huang, Jun-Yan Liu, Xiao-Bo Xia, Ying-Zi Liu, Xi Li, Ji-Ye Yin, Jing-Bo Peng, Lin Wu, Wei Zhang, Hong-Hao Zhou, Zhao-Qian Liu

**Affiliations:** ^1^Department of Clinical Pharmacology, Xiangya Hospital, Central South University, Changsha, China; ^2^Hunan Key Laboratory of Pharmacogenetics, Institute of Clinical Pharmacology, Central South University, Changsha, China; ^3^National Clinical Research Center for Geriatric Disorders, Xiangya Hospital, Central South University, Changsha, China; ^4^Department of Ophthalmology, Xiangya Hospital, Central South University, Changsha, China; ^5^Department II of Thoracic Medicine, Hunan Cancer Hospital, Changsha, China

**Keywords:** circular RNA, competing endogenous RNA, cisplatin sensitivity, non-small cell lung cancer, nucleotide excision repair signaling pathway

## Abstract

**Background:** Despite great advances in the diagnosis and treatment of non-small cell lung cancer (NSCLC), early diagnosis remains a challenge because patients usually have advanced lung cancer at the time they are diagnosed. The limited efficacy of conventional chemotherapy is another major problem in the treatment of NSCLC. Based on a published set of sequencing data, we find that hsa_circ_0001946 is a circRNA molecule with a significantly different expression level in three cell lines (human normal lung fibroblasts cell line MRC-5, human NSCLC cell line A549, cisplatin-resistant cell line A549/DDP), NSCLC tissues and paired adjacent normal tissues. We believe that hsa_circ_0001946 may have an effect on the progression of NSCLC and its sensitivity to cisplatin.

**Methods:** We focused on investigating the circular RNA, hsa_circ_0001946. RNA interference of hsa_circ_0001946 was carried out in A549 cell lines to determine the effect of reduced hsa_circ_0001946 expression on lung cancer progression and was analyzed by Cell Counting Kit-8 (CCK-8), 5-ethynyl-20-deoxyuridine, clone formation, Hoechst, wound healing, and transwell assays. The nucleotide excision repair (NER) signaling pathway was identified by Kyoto Encyclopedia of Genes and Genomes (KEGG) pathway analysis. Moreover, cellular responses to cisplatin were assessed through CCK-8 and flow cytometry assays. Western blot analysis and host-cell reactivation assay were used to determine the effect of hsa_circ_0001946 on NER signaling.

**Results:** In this study, we found that the reduced expression of hsa_circ_0001946 promoted the viability, proliferation, migration, and invasion of NSCLC cells, as well as inhibition of cell apoptosis. Our findings suggest that hsa_circ_0001946 can affect the sensitivity of NSCLC cells to the chemotherapeutic drug cisplatin via modulation of the NER signaling pathway.

**Conclusions:** Our study demonstrated the role of hsa_circ_0001946 in NSCLC pathogenesis, development, and chemosensitivity, and suggests that hsa_circ_0001946 may serve as a novel biomarker for the diagnosis and prediction of platinum-based chemosensitivity in patients with NSCLC.

## Introduction

Lung cancer is a deadly disease associated with the highest morbidity and mortality in the world (1). According to a report released by the World Health Organization in 2018, there are 18.1 million newly diagnosed cancer cases and 1.8 million lung cancer-related deaths worldwide each year. Based on the statistical data released by the China Cancer Center in 2016, 733,000 out of the 4.29 million new-onset cancer patients diagnosed in the previous year received a diagnosis of lung cancer. Furthermore, 610,000 out of 2.8 million cancer-related deaths are caused by lung cancer, making it a “deadly cancer.”

Lung cancer is histologically divided into two main types: small cell lung cancer and non-small cell lung cancer (NSCLC). NSCLC is the most common type of lung cancer, accounting for ~80–85% of all lung cancer cases (2). Chemotherapy, the main treatment for lung cancer, has been applied in more than 90% of patients, with platinum-based chemotherapy being the primary treatment approach (3, 4). However, the efficacy of conventional chemotherapy varies among patients depending on their hypersensitivity, non-sensitivity, or gradual decrease in sensitivity to chemotherapeutic drugs. Drug resistance has become a major challenge in the clinical treatment of NSCLC (5); however, its underlying molecular mechanisms are not yet well understood.

DNA is the optimal target for cisplatin. While cisplatin can kill tumor cells by inducing DNA double-strand breaks, tumor cells can also develop resistance to cisplatin by enhancing DNA repair ability (6). DNA repair ability is the main factor that determines the cisplatin resistance. Different DNA repair ability will affect the efficacy and toxic side effects of cells on cisplatin (7). Nucleotide excision repair (NER) is considered to be the most closely related pathway to platinum-based drug resistance (8). The NER pathway is mainly responsible for repairing DNA cross-linking damage caused by cisplatin, cells with decreased NER activity are highly sensitive to cisplatin (9).

Circular RNA (circRNA) is a class of novel RNA with a closed loop that distinct from traditional linear RNA. CircRNAs exert their biological functions through different mechanisms, such as microRNA sponges, transcriptional regulators, interaction with RNA-binding proteins, and translation into proteins (10). The expression of circRNA is frequently dysregulated in cancer. Accumulating evidences suggest that circRNAs play a critical role in the occurrence, development, metastasis, recurrence, therapeutic effects, and prognosis of various cancer types (11–13). It has been reported that circRNA mitochondrial tRNA translation optimization 1 inhibits hepatocellular carcinoma cell proliferation by competitively binding with microRNA-9 (miR-9) (14), and circLARP4 regulates LARP4 expression by competitively binding with miR-424-5p in gastric cancer (15). A previous study showed that circHIPK3 could potentially act as an miR-558 sponge to inhibit heparanase expression in bladder cancer (16). More recently, another study reported that circCCDC66 promotes cell proliferation and metastasis in colon cancer (17). circRNAs also significantly influence various other types of cancer, including gliomas (18), oral cancer (19), breast cancer (20), and hypopharyngeal squamous cell carcinoma (21).

Based on a set of published sequencing data (22), we find that hsa_circ_0001946 is a circRNA molecule closely related to lung cancer with a significantly different expression level in MRC-5 cell line, A549 cell line, A549/DDP cell line, NSCLC tissues, and paired adjacent normal tissues. Our study revealed that hsa_circ_0001946 inhibits proliferation, promotes apoptosis, and mediates cisplatin sensitivity by regulating the nucleotide excision repair (NER) signaling pathway in A549 cell line. These results provide the basis for further studies on the diagnosis, treatment, and functional research of hsa_circ_0001946 in NSCLC.

## Materials and Methods

We obtained 43 NSCLC tissues and paired adjacent non-tumor lung tissues from patients who underwent surgery. In compliance with the World Medical Association International Medical Ethics, all patients provided written informed consent at the time of surgery for the donation of their tissues for this study. All experimental protocols were approved by the Ethics Committee of Xiangya School of Medicine, Central South University, Changsha, China (registration No. CTXY-110008-3). All tissues were immediately frozen in liquid nitrogen after resection until further use.

### Cell Lines Selection Criteria, Cell Culture and Transfection

The aim of our study was to investigate the effect of circRNA on the progression of NSCLC and cisplatin sensitivity. Therefore, we selected human normal lung fibroblasts cell line MRC-5, human NSCLC cell line A549 and cisplatin-resistant cell line A549/DDP for experiments. MRC-5, A549 and A549/DDP cell lines were purchased from the Chinese Academy of Sciences (Shanghai, China). Cells were cultured in RPMI 1,640 medium (Gibco, Gaithersburg, MD) containing 10% fetal bovine serum (FBS; 10099-141; Gibco) and incubated at 37°C in a humidified atmosphere of 5% CO_2_. A549/DDP cells were cultured in a medium supplemented with 2 mg/L cisplatin (P4394; Sigma-Aldrich, St. Louis, MO) to maintain their drug-resistant phenotype. To knockdown hsa_circ_0001946 expression, hsa_circ_0001946-targeting small interfering RNAs (siRNAs) were synthesized by RiboBio (Guangzhou, China) and transfected into cells using Lipofectamine 2000 (Invitrogen, Carlsbad, CA) according to the manufacturer's instructions. Cells were incubated for 24–48 h before the next experiment.

### RNA Extraction, Ribonuclease R (RNase R) Treatment, and Real-Time Quantitative Reverse Transcription Polymerase Chain Reaction (qRT-PCR)

Total RNA was isolated using TRIzol reagent (Invitrogen) according to the manufacturer's instructions and reverse-transcribed into cDNA using random primers. RNase R treatment was carried out, and 1 μg RNA was incubated for 20 min at 37°C with 1 μl RNase R (20U/ul) or without RNase R (Epicenter, Madison, WI). The expression of circRNAs was assessed by qRT-PCR using SYBR Premix Dimer Eraser assay kit and divergent primers (Biosune, Shanghai, China). QRT-PCR was performed on the LightCycler® 480 PCR system (Roche, Basel, Switzerland).

### Genomic DNA (gDNA) Extraction, End-Point PCR and Sanger Sequencing

Genomic DNA was isolated using QIAamp DNA Mini Kit (QIAGEN, Dusseldorf, Germany) according to the manufacturer's instructions. End-point PCR amplification of cDNA and gDNA templates using HieffTM PCR Master Mix (Yeasen, Shanghai, China) according to the manufacturer's protocol by adding divergent primers and convergent primers, respectively. We performed 30 cycles of end-point PCR. End-point PCR products were examined by UV irradiation after separated in 2% agarose gels. Sanger sequencing following end-point PCR was performed by Biosune biotechnology company (Shanghai, China).

### RNA Fluorescence *in situ* Hybridization (FISH)

The specific probes for hsa_circ_0001946 were designed and synthesized by BersinBio (Guangzhou, China), whose sequences are listed in Table 1. RNA FISH assay was performed using the FISH detection kit (BersinBio) according to the manufacturer's instructions. Images of cells were captured by a fluorescence inverted microscope (E200; Nikon, Tokyo, Japan).

**Table 1:** The siRNAs target sequences and FISH probes sequences of hsa_circ_0001946.

**Type**	**Sequences**
SiRNA1	TCTGCAATATCCAGGGTTT
SiRNA2	GTCTGCAATATCCAGGGTT
FISH probes	TGCCATCGGAAACCCTGGATATTGCAGACACTGGAAGACCTGAAT

### RNA Immunoprecipitation (RIP)

RIP assay was performed using EZMagna RIP Kit (Millipore, Billerica, MA) according to the manufacturer's instructions. A549 cell lysates were incubated with anti-rabbit IgG or anti-rabbit AGO_2_ antibodies (Abcam, Cambridge, MA) with rotation at 4°C overnight. Next, the expression of circRNAs was determined by qRT-PCR.

### Cell Viability, Proliferation, and Apoptosis Assays

Cell viability and proliferation were measured by Cell Counting Kit-8 (CCK-8) and 5-ethynyl-20-deoxyuridine (EDU) incorporation assays, respectively. After transfection siRNAs for 24 h, cells were seeded in 96-well plates (1,000 cells/well) and subjected to CCK-8 (Biotool, USA) or EDU (Cat.No: C10310; RiboBio) assays, according to the manufacturer's instructions. The absorbance of the CCK-8 solution was measured at 450 nm using an Eon™ plate reader (BioTek Instruments, Winooski, VT). EDU incorporation in the cells was visualized with the Leica DMI3000 B inverted microscope (Leica, Wetzlar, Germany). Cell apoptosis was evaluated by Hoechst assay (RiboBio) and flow cytometry using an Annexin V-FITC Apoptosis Detection kit (Beyotime, Beijing, China) following the manufacturer's instructions.

### Cell Clone Formation, Migration, and Invasion Assays

For clone formation assay, 1,000 cells were seeded into 6-well plates (1,000 cells/well; Corning, Inc., Corning, NY) and incubated for 12 days in medium containing 10% FBS. The cell clusters were stained with crystal violet according to the manufacturer's instructions. Wound healing assay and transwell assay (without Matrigel coating) were performed to evaluate cell migration. Transwell assay (with Matrigel coating) (BD Biosciences, Franklin Lakes, NJ) was performed to assess cell invasion. For wound healing assay, the cells were photographed 24 and 48 h after they were scratched with a pipette tip. For transwell assay, 3 × 10^5^ cells (without Matrigel) or 10 × 10^5^ cells (with Matrigel) in serum-free medium were seeded into the upper chamber. After incubation for 24 h at 37°C in a humidified atmosphere of 5% CO_2_, the number of cells that migrated to the bottom wells was counted after staining with a solution containing 0.1% crystal violet and 20% methanol.

### Western Blot Analysis

After transfection for 48 h, cell lysates were prepared using RIPA lysis buffer containing proteinase inhibitor (Roche). Total cell lysates were separated by sodium dodecyl sulfate-polyacrylamide gel electrophoresis and then transferred onto polyvinylidene fluoride (PVDF) membranes (Millipore). After 2 h of blocking, PVDF membranes were incubated with antibodies against β-actin (Sigma-Aldrich), AKT, P-AKT, Bcl-2, Bax, caspase 3, cleaved caspase 3, p53, EGFR, replication protein A 32 (RPA32) (Wanleibio, Shenyang, China), xeroderma pigmentosum complementation group A (XPA), xeroderma pigmentosum complementation group C (XPC), RPA70, radiation sensitive 23 homolog B (Rad23B) (Proteintech, Rosemont, IL), RPA14 (Omnimabs, Alhambra, CA), RPA70 (Abcam, Cambridge, UK), and ERCC excision repair 1 (ERCC1) (Cell Signaling Technology, Boston, USA) overnight at 4°C. Next, membranes were incubated with appropriate anti-rabbit IgG or goat anti-mouse IgG secondary antibodies at room temperature for 2 h. Protein bands were detected using the ChemiDoc XRS+ imaging system (Bio-Rad Laboratories, Hercules, CA).

### Drug Sensitivity Assay

First, cells were seeded in 96-well plates (4,000 cells/well) and incubated overnight at 37°C in a humidified atmosphere of 5% CO_2_. Then, cells were cultured with different concentrations of cisplatin for 48 h at 37°C, followed by incubation with CCK-8 solution according to the manufacturer's instructions. Drug susceptibility was detected by measuring the absorbance at 450 nm using an Eon plate reader. Different drug concentrations for the calculation of the half-maximal inhibitory concentration (IC_50_) were estimated from the relative survival curves. Cisplatin was obtained from Sigma-Aldrich (P4394).

### Host-Cell Reactivation Assay

The firefly luciferase assay-based host cell reactivation (HCR) assay for detecting NER activity. Before transfection, irradiation of pCMV plasmid are illuminated by 254 nm UV on ice using a Stratalinker UV Crosslinker (Stratagene). UV-induced damages were validated by Agarose Gel Electrophoresis (AGE) experiment. Use pRL-TK plasmid encoding renal luciferase as a control. Twenty four hours after transfection siRNA and two plasmids, firefly and renilla luciferase are activity detected by Dual-Luciferase Reporter Assay System (Promega) on a luminometer (Berthold).

### Bioinformatics and Kyoto Encyclopedia of Genes and Genomes (KEGG) Pathway Enrichment Analysis

The target miRNAs of hsa_circ_0001946 were predicted by TargetScan (23), miRanda (24), RNAhybrid (25), and RegRNA 2.0 (26). To establish the hsa_circ_0001946-miRNAs-mRNAs network, we searched for target mRNAs via miRDB (27), and DIANA-microT (28). The graph of the hsa_circ_0001946-miRNAs-mRNAs axis was illustrated using Cytoscape version 3.6.0. Functional enrichment analysis tool (FunRich) was utilized to perform KEGG pathway enrichment analysis (29).

### Statistical Analysis

The SPSS 22.0 software was used for all statistical analyses.

## Results

### Screening of hsa_circ_0001946

**Figure 1:**
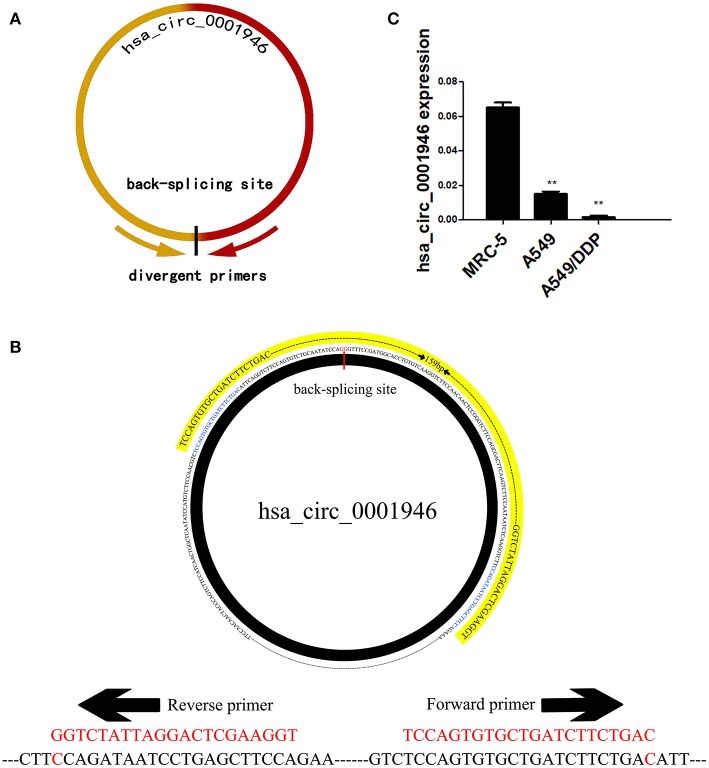
Screening of hsa_circ_0001946 **(A)**. The design principles for primers **(B)**. The design schematic for hsa_circ_0001946 primers **(C)**. The expression level of hsa_circ_0001946 in MRC-5, A549, and A549/DDP cell lines (All data are presented as the mean ± SEM, *P < 0.05, **P < 0.01, ***P < 0.001).

Based on the RNA sequencing data mentioned above, we selected 10 circRNAs with the highest expression in lung tissue, and then measured their expression levels in human normal lung fibroblasts cell line MRC-5, human NSCLC cell line A549, cisplatin-resistant cell line A549/DDP. We designed a pair of divergent primers for the circRNA that span the back-splice site (Figure 1A, Table 2). Hsa_circ_0001946 is a circRNA molecule with a significantly different expression level in three cell lines. The cDNA of hsa_circ_0001946, around 159bp upstream and downstream from the back-splice site, was amplified (Figure 1B). Hsa_circ_0001946 was highly expressed in MRC-5 cell line compared with that in A549 cell line. Interestingly, its expression was significantly lower in A549/DDP cells compared with that in A549 cells (Figure 1C).

**Table 2:** The 10 circRNAs with the highest expression in lung tissue based on RNA sequencing data.

**Gene symbol**	**circRNA**	**Position**	**Primer sequences**
HIPK3	hsa_circ_0000284	chr11:33307958-33309057	F: 5′ TATGTTGGTGGATCCTGTTCGGCA 3′
			R: 5′ TGGTGGGTAGACCAAGACTTGTGA 3′
ZNF609	hsa_circ_0000615	chr15:64791491-64792365	F: 5′ AGAGGAAGGGGAGAATGAGTG 3′
			R: 5′ GCTCAAGGACATCTTAGAGTCAACG 3′
CDR1	hsa_circ_0001946	chrX:139865339-139866824	F: 5′ TCCAGTGTGCTGATCTTCTGAC 3′
			R: 5′ TGGAAGACCCGGAGTTGTTG 3′
SMARCA5	hsa_circ_0001445	chr4:144464661-144465125	F: 5′ AGATGGGCGAAAGTTCACTTAG 3′
			R: 5′ CCATCGATATCTTCATCAGTGATC 3′
UBXN7	hsa_circ_0001380	chr3:196118683-196129890	F: 5′ TCTGGTTCCACCAGTATTTCCT 3′
			R: 5′ CCTTCTGTATTATAGTTCGGCAAG 3′
N4BP2L2	hsa_circ_0000471	chr13:33091993-33101669	F: 5′ ATACCTGTACCCATCTTGATGGT 3′
			R: 5′ ATGAATTCAGTGGAACCATCAC 3′
ESYT2	hsa_circ_0001776	chr7:158552176-158557544	F: 5′ CTCTGCTTTGGAAGATTTGGTTG 3′
			R: 5′ AAGCCAACGATGGTCTTTCCT 3′
FBXW7	hsa_circ_0001451	chr4:153332454-153333681	F: 5′ TACCCTCTGACCCAGTAACTCCAC 3′
			R: 5′ TAGTATTGTGGACCTGCCCGTT 3′
LIFR	hsa_circ_0072309	chr5:38523520-38530768	F: 5′ TCTTTTATTGTCCACCATCCAGG 3′
			R: 5′ TTCCACACCGCTCAAATGTTATC 3′
SLC8A1	hsa_circ_0000994	chr2:40655612-40657444	F: 5′ GTGAAAGACTTAATCGCCGCAT 3′
			R: 5′ CCATATAAAACCATCGAAGGGACT 3′

### Characterization of hsa_circ_0001946

**Figure 2:**
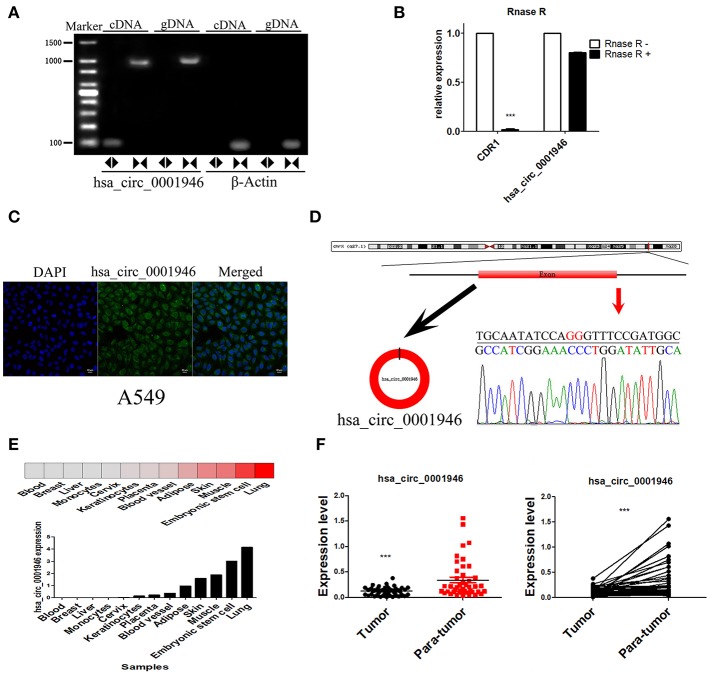
Characterization of hsa_circ_0001946 and its parental gene CDR1 **(A)**. Divergent primers were used to amplify hsa_circ_0001946 in cDNA but not gDNA. β-actin was used as negative control **(B)**. After RNase R treatment, the expression levels of CDR1 and hsa_circ_0001946 were determined by qRT-PCR **(C)**. RNA FISH assay was used to detect the localization of hsa_circ_0001946 in A549 cells **(D)**. A schematic diagram of the genomic location and splicing pattern of hsa_circ_0001946. The back-splicing site was verified by Sanger sequencing **(E)**. The expression of hsa_circ_0001946 in various human organs **(F)**. The expression level of hsa_circ_0001946 in 43 NSCLC tissues and matched adjacent non-tumor tissues (All data are presented as the mean ± SEM, *P < 0.05, **P < 0.01, ***P < 0.001).

According to CIRCBASE database annotation, hsa_circ_0001946 is transcribed from cerebellar degeneration related protein 1 (CDR1), hsa_circ_0001946 is located at chrX: 139865339-139866824 and consists of one exon, with a length of 1485nt. We performed end-point PCR assay, RNase R treatment and FISH assay to verify hsa_circ_0001946 expression. We did not use RNase R treatment of cellular RNA, but used divergent primers and convergent primers for end-point PCR assay. End-point PCR assay performed with divergent primers indicated that hsa_circ_0001946 was expressed in A549 cells cDNA (104bp) but not genomic DNA (gDNA). End-point PCR assay performed with convergent primers indicated that hsa_circ_0001946 was expressed in cellular cDNA (104bp) and gDNA (1036bp) (Figure 2A), and also confirmed that compared to CDR1 mRNA, hsa_circ_0001946 was resistant after RNase R treatment of cellular RNA (Figure 2B). The results of RNA FISH assay showed that hsa_circ_0001946 was mainly localized in the cytoplasm (Figure 2C). Moreover, Sanger sequencing of the end-point PCR products amplified by divergent primers further confirmed the back-splicing site of hsa_circ_0001946 (Figure 2D). By using CIRCNET database annotation, we evaluated the expression of hsa_circ_0001946 in various tissues, the result was consistent with RNA sequencing data, and it was highly expressed in the lung tissue (Figure 2E). Then, we measured the expression level of hsa_circ_0001946 in NSCLC and paired adjacent normal tissues, qRT-PCR results showed that hsa_circ_0001946 expression was downregulated in 43 NSCLC tissues (Figure 2F), which have been listed in Table 3. These results confirmed the existence of hsa_circ_0001946, and suggest that it may be associated with the development of lung cancer.

**Table 3:** Correlation between hsa_circ_0001946 expression and clinicopathological characteristics in 43 NSCLC patients.

**Clinical and pathological variables**	**N (%)**	**hsa_circ_0001946 expression levels**		**Mean ± SD**	***P*-value**
		**High expression**	**Low expression**		
**AGE (YEARS)**					
<60	21(48.84)	4	17	0.12 ± 0.07	0.9631
≥60	22 (51.16)	3	19	0.12 ± 0.09	
**GENDER**					
Male	40 (93.02)	7	33	0.12 ± 0.08	0.8673
Female	3(6.98)	0	3	0.12 ± 0.08	
**SMOKING STATUS**					
Smoker	32 (74.42)	6	26	0.13 ± 0.07	0.8231
Non-smoker	11(25.58)	1	10	0.12 ± 0.09	
**CLINICAL STAGE**					
I-II	20 (46.51)	5	15	0.12 ± 0.09	0.9735
III-IV	23 (53.49)	2	21	0.12 ± 0.08	
**DIFFERENTIATION**					
high	10 (23.26)	0	10	0.11 ± 0.11	0.8776
Moderately	22 (51.16)	4	18	0.13 ± 0.07	
Poorly	11(25.58)	3	8	0.13 ± 0.07	
**LYMPH NODE METASTASIS**					
Yes	19 (44.19)	2	17	0.13 ± 0.08	0.4791
No	24 (55.81)	5	19	0.11 ± 0.08	

### Hsa_circ_0001946 Plays a Tumor Suppressive Role in Lung Cancer Cells

**Figure 3:**
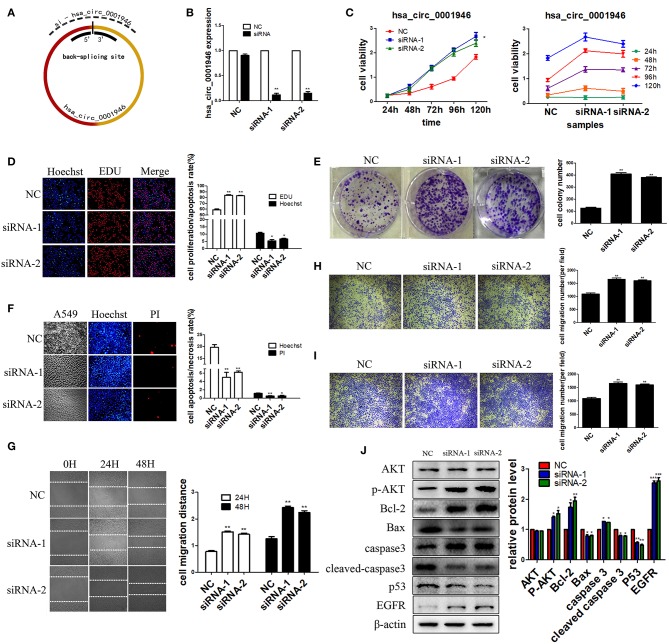
hsa_circ_0001946 plays a tumor suppressive role in NSCLC cells. We performed a series of experiments following transfection **(A)**. The design principles for hsa_circ_0001946 siRNAs **(B)**. The knockdown efficiency of siRNAs **(C)**. The viability of cells was evaluated by CCK-8 assay. Cell proliferation was assessed by EDU **(D)** and clone formation assays **(E,F)**. Apoptosis of cells was detected by Hoechst assay. The migratory capability of cells was assessed by wound healing assay **(G)** and transwell assay (without Matrigel) **(H,I)**. The invasive capability of cells was determined by transwell assay (with Matrigel) **(J)**. Western blot analysis: proteins were isolated from transfected cells as indicated (All data are presented as the mean ± SEM, *P < 0.05, **P < 0.01, ***P < 0.001).

We selected A549 as the experimental cell line. To evaluate the possible role of hsa_circ_0001946 in lung cancer, we constructed two specific siRNA vectors at the back-splicing site (Figure 3A). QRT-PCR results indicated that transfection of siRNA vectors significantly downregulated the endogenous hsa_circ_0001946 expression level (Figure 3B). Subsequently, the roles of hsa_circ_0001946 in lung cancer cell viability, proliferation, apoptosis, invasion, and migration were explored. Knockdown of hsa_circ_0001946 expression noticeably enhanced cell viability and proliferation (Figures 3C–E). In addition, downregulation of hsa_circ_0001946 expression led to a decrease in the number of apoptotic cells (Figure 3F), and remarkably increased the wound healing ability of lung cancer cells (Figure 3G). Furthermore, downregulated hsa_circ_0001946 expression enhanced the migratory and invasive capabilities of lung cancer cells (Figures 3H,I). Compared to that in negative control group, western blot analysis showed that downregulated hsa_circ_0001946 expression significantly reduced the apoptosis-related protein expression and increased the proliferation-related protein expression in lung cancer cells (Figure 3J). In summary, our data suggests that hsa_circ_0001946 plays a tumor suppressive role in lung cancer cells.

### Prediction of hsa_circ_0001946 Signaling Pathway

**Figure 4:**
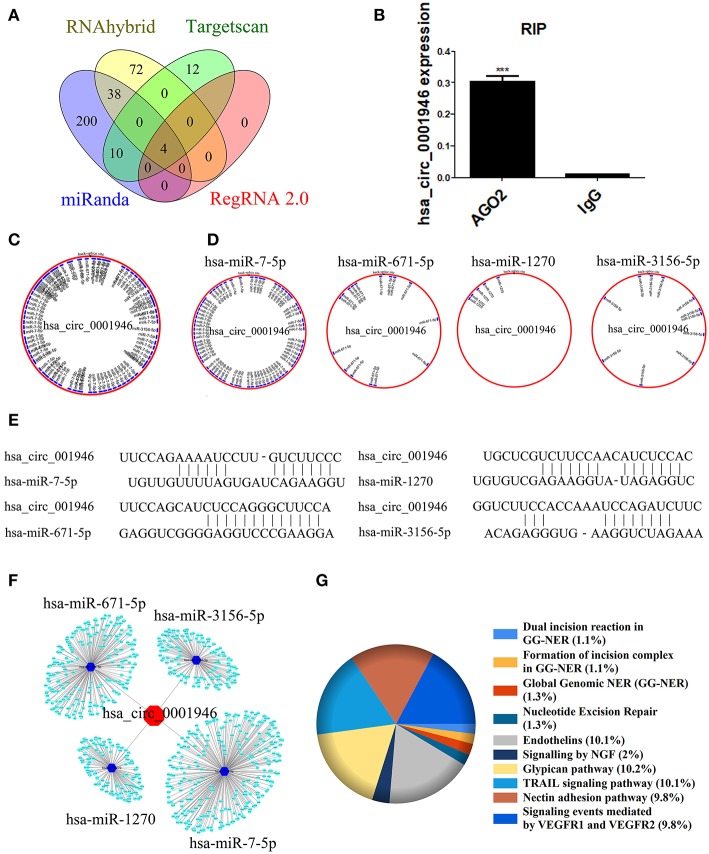
Bioinformatics analysis was used to predict the hsa_circ_0001946 signaling pathway **(A)**. Venn diagram of the overlapping parts of the four sets of databases. Four miRNAs in total were common to all databases sets **(B)**. RIP assay indicating that hsa_circ_0001946 was substantially accumulated in the AGO_2_ pellet **(C)**. Four miRNA binding sites on hsa_circ_0001946 (merger) **(D)**. Four target miRNA binding sites on hsa_circ_0001946 (independence) **(E)**. Four target miRNA binding sequence schematic graph on hsa_circ_0001946 **(F)**. The network of hsa_circ_0001946-miRNAs-mRNAs axis **(G)**. KEGG pathway enrichment analysis for hsa_circ_0001946 pathway (All data are presented as the mean ± SEM, *P < 0.05, **P < 0.01, ***P < 0.001).

There are a number of miRNAs biding sites on most circRNAs. circRNAs can be enriched by miRNAs as competing endogenous RNAs (ceRNAs) and suppress the activity of miRNAs (10, 30). We predicted signaling pathways to explore the function of hsa_circ_0001946. We used miRanda, RNAhybrid, Targetscan, and RegRNA 2.0 database to determine the target miRNAs of hsa_circ_0001946. The intersection of the four databases consists of four elements, which is hsa-miR-7-5p, hsa-miR-671-5p, hsa-miR-1270 and hsa-miR-3156-5p (Figure 4A). Next, RIP for AGO_2_ and lgG in A549 cells was performed, and the results indicated that hsa_circ_0001946 was significantly accumulated in the AGO_2_ pellet (Figure 4B). In addition, we plotted the binding sites and binding sequence schematic graph of the four target miRNAs on hsa_circ_0001946 (Figures 4C–E). Next, we predicted the target protein of the four miRNAs via miRDB and DIANA-microT database. Furthermore, we selected the intersection of the two databases for KEGG pathway analysis (Supplementary Table 1). The network of hsa_circ_0001946-miRNAs-mRNAs axis was illustrated by Cytoscape (Figure 4F), and the KEGG pathway enrichment analysis was delineated using FunRich (Figure 4G). Interestingly, pathway prediction analysis showed that the target proteins identified were associated with the NER signaling pathway. As the most important DNA repair system in organisms, the main genes of the NER signaling pathway include XPA, XPC, Rad23B, RPA14, RPA32, RPA70, and ERCC1. According to a previous report, the NER signaling pathway is related to the cisplatin sensitivity of lung cancer cells (31). Our results suggest that hsa_circ_0001946 might function as a ceRNA, while regulating the sensitivity of lung cancer cells to cisplatin through the NER signaling pathway.

### Hsa_circ_0001946 Mediates Cisplatin Resistance via the NER Signaling Pathway

**Figure 5:**
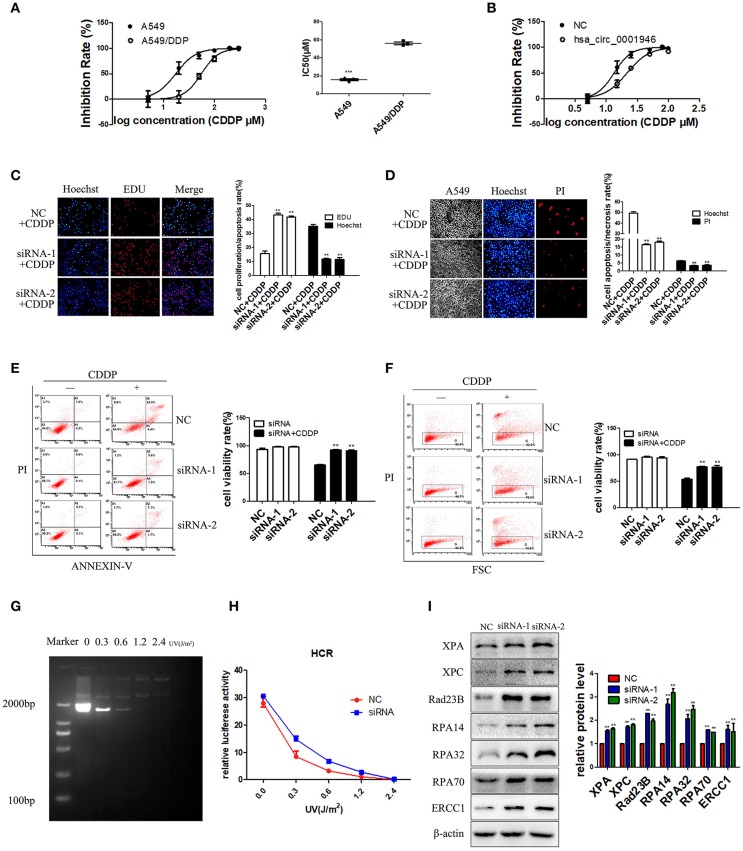
hsa_circ_0001946 mediated cisplatin resistance via the NER signaling pathway **(A)**. The IC_50_ values of cisplatin in A549 and A549/DDP cell lines **(B)**. The IC_50_ value of cisplatin after transfection of A549 cells with hsa_circ_0001946 siRNA. The hsa_circ_0001946 group exhibited a higher IC_50_ value of cisplatin than the negative control group **(C)**. Cell proliferation was evaluated by EDU assay after siRNA transfection and treatment with 15 μM cisplatin **(D)**. Apoptosis of cells was assessed by Hoechst assay after siRNA transfection and treatment with 15 μM cisplatin **(E)**. Representative flow cytometry results showing that the effects of hsa_circ_0001946 on cisplatin decreased cell apoptosis in the A549 cell line **(F)**. Cell viability was also evaluated by dye exclusion assay using flow cytometry after siRNA transfection and treatment with 15 μM cisplatin **(G)**. HCR analysis of UV damaged pCMV plasmid **(H)**. The damaged pCMV plasmids were then transfected into scrambled control or hsa_circ_0001946 silenced cells followed by analysis of luciferase activity. HCR results showed increased DNA damage repair in siRNA transfection group **(I)**. The expression of the NER pathway-associated proteins, XPA, XPC, Rad23B, RPA14, RPA32, RPA70, and ERCC1 was detected by western blotting after siRNA transfection (All data are presented as the mean ± SEM, *P < 0.05, **P < 0.01, ***P < 0.001).

We next explored whether hsa_circ_0001946 could affect cisplatin resistance and increase the activity of the NER signaling pathway in lung cancer cells. We evaluated the IC_50_ value of cisplatin to identify the resistance index of A549 and A549/DDP cells. The IC_50_ value of cisplatin in A549 cells was significantly lower than that in A549/DDP cells (Figure 5A). We also found that downregulation of hsa_circ_0001946 expression increased cisplatin resistance in A549 cells (Figure 5B). Furthermore, the effect of cisplatin on the proliferation and apoptosis of lung cancer cells was detected by EDU, Hoechst, and flow cytometry assays after downregulation of hsa_circ_0001946 expression. The results demonstrated that cisplatin treatment of lung cancer cells with downregulated hsa_circ_0001946 expression increased cell proliferation (Figure 5C), and reduced cell apoptosis compared to the negative control group (Figures 5D–F). Next, we used HCR assay and western blot analysis to determine whether downregulation of hsa_circ_0001946 expression caused activation of the NER signaling pathway. We used HCR assay to analyze whether knockdown of hsa_circ_0001946 expression affected NER activity. For this purpose, pCMV plasmid containing luciferase reporter was UV irradiated in order to generate DNA adducts which were confirmed by PCR analysis (Figure 5G). The pRL-TK plasmid and damaged pCMV plasmids were then transfected into hsa_circ_0001946-silenced or scrambled control cells followed by analysis of luciferase activity (Figure 5H). Western blot showed that the expression of XPA, XPC, Rad23B, RPA14, RPA32, RPA70, and ERCC1 was up-regulated after knockdown of hsa_circ_0001946 expression (Figure 5I). These results demonstrated that knockdown of hsa_circ_0001946 expression activated the NER signaling pathway, which in turn reduced cisplatin sensitivity and increased DNA repair ability. These data further confirmed that hsa_circ_0001946 influences the cisplatin resistance of lung cancer cells through the NER signaling pathway.

## Discussion

Lung cancer is a fatal cancer with high morbidity and mortality, and its treatment is challenged by issues with early diagnosis and resistance to conventional therapy. Cisplatin is a chemotherapeutic drug widely used in the clinical treatment of lung cancer (4, 32). However, endogenous and acquired drug resistance limits its clinical efficacy (33). Therefore, it is of clinical significance to identify early diagnostic biomarkers for lung cancer and novel targets for enhancing cisplatin chemosensitivity.

In this study, we found that hsa_circ_0001946 was highly expressed in lung tissues, and its parental gene was associated with the prognosis of lung cancer. We measured the expression level of hsa_circ_0001946 in 43 NSCLC tissues and matched adjacent non-tumor tissues, and our data showed that the expression of hsa_circ_0001946 was downregulated in NSCLC tissues. Subsequently, hsa_circ_0001946 expression level was measured in three different cell lines, and results showed that MRC-5 cells expressed the highest level of hsa_circ_0001946, followed by A549 and A549/DDP cells. Next, we knocked down the expression of hsa_circ_0001946 in A549 cells and evaluated its effect on cell viability, proliferation, apoptosis, migration, and invasion. Our results suggest that hsa_circ_0001946 acted as a tumor suppressor in lung cancer cells. We predicted the downstream miRNAs and target mRNAs of hsa_circ_0001946 via bioinformatics analysis, as well as the possible pathways through KEGG pathway analysis. Our data suggest that hsa_circ_0001946 might affect the sensitivity of lung cancer cells to cisplatin through the NER signaling pathway, which was experimentally validated by EDU, Hoechst, flow cytometry, and western blot assays.

Until recently, the biological functions of circRNAs were not well understood since only a few circRNAs had been explored. Herein, we reported the low expression of hsa_circ_0001946 in human lung cancer tissues and cell lines. Besides, hsa_circ_0001946 expression was significantly downregulated in cisplatin-resistant A549/DDP cells compared to that in parental A549 cells. Functionally, we found that downregulation of hsa_circ_0001946 expression promoted cell viability, proliferation, migration, invasion, reduced apoptosis and cisplatin sensitivity. Mechanistically, hsa_circ_0001946 might influence cisplatin sensitivity in lung cancer patients by regulating the NER signaling pathway through sponging four predicted miRNAs (hsa-miR-7-5p, hsa-miR-671-5p, hsa-miR-1270 and hsa-miR-3156-5p), as demonstrated by bioinformatics analysis, western blotting and HCR assay results.

**Figure 6:**
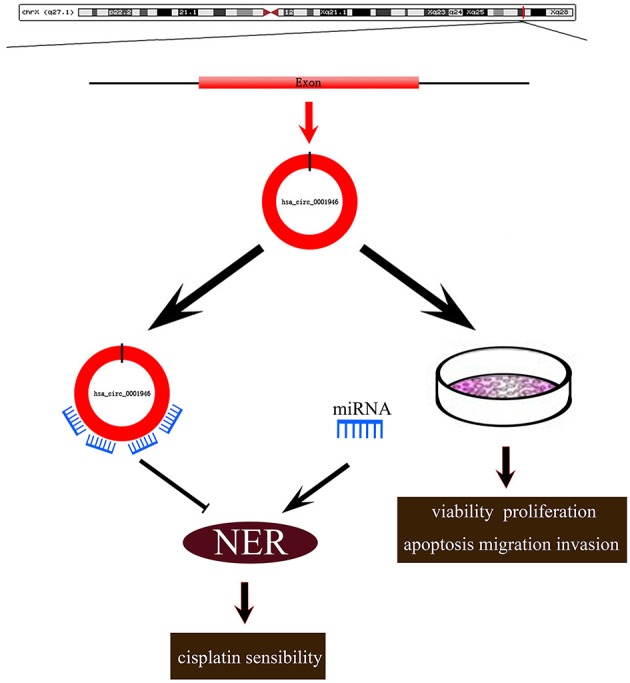
Roles of hsa_circ_0001946 in lung cancer. Hsa_circ_0001946 not only directly regulated the proliferation, apoptosis, migration, invasion of lung cancer cells, but also influenced cisplatin resistance through the NER signaling pathway.

In conclusion, our findings revealed the role of hsa_circ_0001946 in NSCLC development, and chemosensitivity (Figure 6). Our results indicated that hsa_circ_0001946 may serve as a novel biomarker for the survival probability and predicting the chemosensitivity of cisplatin in lung cancer patients.

## Data Availability

Publicly available datasets were analyzed in this study. This data can be found here: a “http://www.targetscan.org/mamm_31/.”

## Ethics Statement

A total of 43 patients with non-small cell lung cancer were enrolled at Xiangya Hospital of Central South University (Changsha, Hunan, China). Our study was approved by the Ethics Committee of Xiangya School of Medicine, Central South University (Registration number: CTXY-110008-2 and CTXY-110008-3). Written informed consent was obtained from all non-small cell lung cancer patients.

## Author Contributions

H-HZ, J-BP, and Z-QL: conceptualization. M-SH: data curation, formal analysis, investigation, writing—original draft, and writing—review and editing. Z-QL and LW: funding acquisition. X-BX: methodology. Y-ZL: resources. J-YL: software. XL and J-BP: supervision. J-YY: validation. WZ: visualization.

### Conflict of Interest Statement

The authors declare that the research was conducted in the absence of any commercial or financial relationships that could be construed as a potential conflict of interest.
