# Supplementary material for: Hsa_circ_0001946 Inhibits Lung Cancer Progression and Mediates Cisplatin Sensitivity in Non-small Cell Lung Cancer via the Nucleotide Excision Repair Signaling Pathway
Source: Front Oncol. 2019 Jun 12;9:508. doi: 10.3389/fonc.2019.00508 (PMC6582772; doi:10.3389/fonc.2019.00508)
Supplement: Supplementary file 1 [file Table_1.DOC]

**Supplementary Table 1.** Summary the target mRNAs of miRNAs

| miRNA name | total | target mRNAs |
| --- | --- | --- |
| hsa-miR-7-5p | 267 | ST8SIA5 CALM3 AGO1 IDS KRTAP5-4 C1orf226 ANKRD12 MTOR ERCC5 OSBPL11 IPO11 IMPG1 ZNF106 PHACTR4 CCT4 ARHGEF33 RYK U2SURP HERPUD2 LECT1 CCSER2 SLC46A2 TGFB2 RBMS3 RPA14 DSEL GALNT3 OGT SEPT8 ANGPTL5 DNAH14 DLEU7 XPD GAL3ST3 EIF2S3 GUCY1A3 MESDC2 CTSB FNDC4 SHANK2 CHP1 KCNA6 CYTH3 RAF1 NUCKS1 CHAMP1 KBTBD2 EIF4E TRIM33 POLI DIRAS1 HCN1 EDAR ATP2B2 MSH3 DPYSL2 RAB11FIP5 RGS21 AK3 PMEL TAB2 CGGBP1 SMARCD1 CAPZA1 CNOT8 KIAA0247 FAM168A DDIT4 CSMD3 DLGAP1 SETD7 ZNF512 ZC3H4 BACE1 STX17 SERP1 GJC1 ELFN2 CRY2 TRPC5 SCN4B NTRK2 PRKCB MIER2 RNF141 NR1H2 SLC38A2 VPS26A SLC6A9 CCDC66 CRLS1 ICA1L SYT6 SEPT10 SEPW1 APOA2 ASXL1 APOL5 DACH1 CAMKK2 ENO2 RAB11FIP4 PAX6 ZBTB22 CDON PLP2 ITPKC FAM168B SPTY2D1 CNNM4 NDST3 EGFR OXR1 STAU2 KIAA0430 CXorf40A NQO1 CBL SKP1 SP1 KIAA2018 PRDM6 FAM53C ZBTB1 EXOSC2 CNPPD1 RIMKLA ZDHHC9 MAPKAP1 HLA-DOB C5orf22 CTSK UBQLN4 FBXL14 MEGF9 TPGS2 CLEC4M RPL15 LTN1 TMEM254 TRMT13 SCAMP5 GGT7 CCDC43 STRN3 CALU NDFIP2 ANKFY1 C9orf84 EDA IRS2 ABCG4 RBFOX1 TPCN1 PDE4D SNCA UBXN2B XPA TREM1 VDAC1 DTYMK SECISBP2L TMEM43 UBLCP1 CCNI2 PHYHIPL ARF4 GLTPD1 NXT2 HS3ST5 PSME3 FAM131B SH3GLB1 LIMD1 SLC4A7 PIK3CD GLI3 RFX7 LRRC8E SEC24A CACNG7 FBXO28 IRS1 SPATA2 ANKRD50 NR4A3 HELLS NOTCH3 CCNT2 RGS7BP REG3G KIF16B PRKD3 WASF3 ZNF395 ZNF609 GABRA6 IGLON5 LEMD3 PLEC SLC38A4 TCF12 SGIP1 FGD6 ATF7 RSBN1 GATA5 TFF3 TSPYL1 STEAP2 LRTM2 CNN3 PAN2 TMEM97 FCHO2 RNF144A RELA XPB QTRTD1 LRRC59 CLASP2 XPO7 XPC PBX3 ACSL4 WAPAL SATB1 POLE4 RB1 COLEC12 ZMAT3 SLIT1 GABRA1 FLRT2 DCSTAMP NFATC2 ESRRG MAP3K9 KDM3B C1orf21 BMP8B RHBDD1 B3GALTL TTC26 PATL1 ZNF425 SLC25A15 ABCA13 CCDC64B FBXL7 BCAP29 RNF20 ATG4A CKAP4 RPS6KB1 SOX6 ZNF275 PARP1 HNRNPUL2 SETD8 KLF4 BLOC1S4 WDR47 ARID4A DCAF12L2 ZFX JADE1 |
| hsa-miR-671-5p | 204 | HNRNPUL1 DBNL ANKS1A SNN FAM83F FAM219A ATOX1 CD209 CFL2 DLG1 LGALSL VAMP2 SYNPR WFIKKN2 RAB12 CEP41 CD34 ACBD6 PDE6A C10orf25 XDH FNDC5 ZNF185 CBLN3 TIPARP CREB3L2 RNF38 SPPL3 GPR68 RPA70 MCU CXCL14 ADARB1 RAB18 TSPYL5 KCNJ10 Rad23 KRT38 TTL SATB2 ZNF189 MYH9 SASH3 KRT9 ANKS1B SPIRE1 CRTC3 UBR7 GPR107 XPG CELF1 SRGAP3 DMBX1 RECQL FAT4 LPAR1 ERCC3 WDR41 EHD3 ANKRD46 ZIM2 SPTBN1 HMGA1 RAG1 EIF5B SYPL2 PI4K2A TET3 THBS1 PCSK1 TNFRSF1B RNF185 DVL3 ATP5SL TRIM67 RORA SLC7A1 SHISA9 FAM110B VSNL1 PSMB2 LRRK2 XPE HIGD1A TBC1D13 SYT9 ARHGAP40 KDM5A FADS1 MAPK10 USP36 MYRIP PRR5L TMEM164 SMCO3 ZNF318 LGALS3BP LDOC1L FGFR2 CALN1 C16orf72 WNT3 ERCC4 TGOLN2 KIT CTBS SMG1 CTNNBIP1 AAK1 KIF1B CYB5D1 RAB6B USB1 MBD2 ARMC8 FUT4 MXD3 SLC36A1 PHC2 RELT SEPP1 PIGZ PPP2R5C SLC30A6 GBP4 RABGAP1L MARCH5 GID4 VPS52 EDEM3 CD47 MACC1 LRRC46 HEPACAM SAMD12 HP1BP3 WIPF2 PAPLN SMAD3 CRAMP1L USP46 RHD SMEK2 C15orf52 VPS45 PCDH20 LIX1L BAHD1 PRPS1 SLC35D1 CBS GALNT10 PACSIN1 KREMEN1 RASL10B NFKB1 NFYA TP53I11 PIP4K2C HIVEP3 MED15 MDM4 SZRD1 GOLPH3L LIN9 MAGEE1 SSR1 TMEM201 SEPT3 CPNE2 C12orf77 DUSP13 TBC1D5 C14orf28 DCAF17 BCR FOXM1 SGK2 PA2G4 EPPIN ACTR2 GSTM5 MBOAT7 CA7 ZNF740 KLF6 HDAC5 CDIP1 C5orf47 ACVR2A PTPRO KCNA7 CSGALNACT1 GRAMD1C DDX39B SPOCK1 SHISA5 PIK3IP1 PRC1 RLF LRRC19 PASD1 KCNN3 XPF |
| hsa-miR-1270 | 113 | SET SLC1A1 MEMO1 GPR155 UNC45B CNDP1 CSNK2A1 RFC5 CELSR2 MUM1L1 HECW2 F8 RIC3 ENTPD3 TUBB CCNYL1 FLRT3 GTDC1 CNTN3 ACTBL2 PLCB1 COPS4 SPATA5 POU2F3 ADCY2 TMEM183A IL1RAP ZFP30 LYSMD1 TCF20 HOXC5 GLI2 DSC2 NUBPL PTPRT MBNL2 SH3BGRL2 UBASH3B EPB41L4B PPP3R1 SHISA3 EDIL3 MED17 CAPRIN1 CSRNP1 PRR23C TNFSF13 DDB2 C1orf101 LGI1 CXXC5 TMLHE KLHDC3 SULT2A1 ANGPTL7 KHDRBS3 OLA1 GLRA3 FBXO42 STOML3 TBC1D30 CBFB C15orf40 TMEM167A C1orf174 DCPS CACNA1E AFP FMO4 CNGB3 SUN2 ERCC1 TESC UTP23 DENND1B ERCC2 RASAL2 ZIC5 NEUROD4 KRTAP4-11 TNFSF12-TNFSF13 IRF2BP2 PIP4K2B COPS2 DGKE FAM120C SEPT11 IKZF5 KCNA4 RPA32 HECW1 DDI2 CEP164 UNC5D PLXNA4 DENND2C SENP5 UEVLD PHF8 BET1L STK4 ZNF621 Rad23B CALCR NR3C1 LEP ARID1B UNC5CL SLITRK2 ATP12A XKR9 CHSY1 LAMB4 |
| hsa-miR-3156-5p | 129 | FKBP5 KIF24 TMPO USP30 IFT74 PLAG1 CCPG1 TMOD2 FLI1 SCN2A MB21D2 MYO1B RTN4 GAPVD1 DMTF1 SCMH1 KLHL2 EXD2 MEIS2 CPEB3 VPS54 CNTN5 DCAF10 GBP5 HSDL2 MTA2 MRPL50 KIAA1217 CITED2 KPNA4 RAP1A KIAA1024 MORF4L1 MORF4L2 PDZRN3 ADCY3 ZMYND11 C3orf58 CDC123 AFF4 PAG1 TRERF1 RPRD1A BCAT1 SGOL1 RASSF2 EFCAB14 FGF13 SLC16A9 WWTR1 CHAC2 KCNV1 PML PPP2R5A SESN3 ZMYM2 NRG3 ING3 KCNJ16 TMEM65 PHF20L1 KIAA1841 ELAVL2 ZC3H12C FAM175B ATXN1 UBE2H POLR3G SDC2 TBC1D15 C9orf72 C3orf38 TMCC1 LHFPL2 ELOVL5 ZFR SESTD1 ARID5B TRAPPC2 FAM118B EPHA7 ABL2 EPM2AIP1 ASCC3 RALGPS2 ZDBF2 RASSF8 PDGFRA HUNK DTNA RRM2B WDR36 HNRNPA2B1 STX11 FAM149B1 ZBTB10 ACBD3 TACC1 RAB27A PTBP3 TMEM170B HSPA12A SNX18 SCRT2 SDF2 CLDN1 FUBP1 STXBP4 SNX31 TNPO1 KATNAL1 XRN1 MGA PSD3 ST18 KCND2 UBE2D1 ZNF711 CDK13 HRH4 HPSE RAB7A AJAP1 MEF2C FAM169A MBNL1 DEPDC1 COPS7B SKAP2 |
